# Rewiring Adipocyte Plasticity: The Redox–Mitochondrial Axis as a Driver of Beige Fat Immunometabolism and Adipose Inflammation

**DOI:** 10.3390/biom16050701

**Published:** 2026-05-09

**Authors:** Faycal Aissa-Brahim, Shuwen Yang, Xunyong Zhou, Zhong Li, Su Bu

**Affiliations:** 1 College of Life Science, Nanjing Forestry University, Nanjing 210037, China; faycalab@njfu.edu.cn (F.A.-B.);; 2 ZhenCui (Jiangsu) Enzyme Technology Development Co., Ltd., Suqian 223600, China; 3 Agricultural and Forestry Products Deep Processing Technology and Equipment Engineering Center of Jiangsu Province, Nanjing Forestry University, Nanjing 210037, China

**Keywords:** obesity, adipocyte, inflammation, mitochondria, immunometabolism

## Abstract

Obesity is a major health crisis that impacts social and economic burdens worldwide, and it is being studied intensively to understand the molecular mechanisms and regulators of this complex condition. Over the past decade, significant insights into adipocyte fate have been gained, demonstrating that adipocyte behavior is influenced by intracellular pathways, systemic mediators, and environmental signals. In this review, we discuss the current understanding of key molecular and functional changes in adipose tissue. We focused on ER–mitochondrial dynamics and their role in beige adipocytes. We examined the biological properties of beige fat cells, including their inflammatory status, redox balance, and regulation of adipocyte transdifferentiation via signaling molecules. Finally, we explored the effects of naturally derived compounds on adipocyte function and their potential to combat obesity using a comprehensive approach. The process of adipocyte beiging induced by coordinated intracellular signaling pathways highlights mechanisms that can reprogram metabolic homeostasis and reduce obesity-related phenotypes.

## 1. Introduction

By 2030, one billion people globally will be living with obesity, underscoring the escalating public health crisis [1]. Central to this is the dysregulation of energy homeostasis, which prompts excessive growth and remodeling of adipose tissue due to surplus energy intake over expenditure [2]. Adipocytes have long been known for their ability to sense and respond to external signals, helping the body store or release nutrients based on its metabolic needs [3]. The defining structural traits of white and brown adipose tissues reflect their specialized functions, and these inherent differences sustain their contrasting yet sometimes overlapping roles in metabolic regulation [4]. Although brown adipose tissue (BAT) constitutes a small fraction of the total adipose mass in humans, it is metabolically active, contributing to energy expenditure through enhanced thermogenesis and oxidative phosphorylation [5]. It is characterized by a multilocular lipid droplet architecture and a high abundance of uncoupling protein 1 (UCP1), a mitochondrial inner membrane protein that dissipates the proton gradient to produce heat rather than adenosine triphosphate (ATP) and regulates non-shivering thermogenesis [6]. Accumulating evidence indicates that under specific physiological or pharmacological stimuli, white adipose tissue (WAT) exhibits remarkable plasticity, giving rise to increased UCP1-expressing, mitochondria-rich beige adipocytes, a process termed ‘browning’ or ‘beigeing’ which functionally resembles classical brown adipocytes in their thermogenic potential [7,8]. Multiple studies have demonstrated the pivotal function of beige adipocytes in alleviating obesity-induced metabolic disturbances [9,10,11]. This transition from energy storage to thermogenic expenditure represents a profound example of metabolic plasticity in living organisms that requires the orchestration of multiple cellular interlocutors [12,13].

This review defines the redox–mitochondrial axis as bidirectional communication between ER stress, mitochondrial dynamics, and metabolic flux, which drives macrophage polarization and white-to-beige adipocyte transdifferentiation. First, we analyzed how ER–mitochondria tethering facilitates the seamless trafficking of UCP1 to the inner mitochondrial membrane, a critical process for maintaining ER and mitochondrial proteostasis during the high protein demand phase of beiging. This subcellular coordination then impacts the immuno-oxidative microenvironment; autophagy, amino acid catabolism, and ROS production act as metabolic rheostats that control inflammasome activation and, subsequently, macrophage polarization. Finally, we synthesized evidence showing that naturally derived bioactive molecules converge on this redox–mitochondrial circuit to promote beiging and reduce fat accumulation across experimental models.

## 2. Implicated Metabolic Pathways and Subcellular Structures in the Reprogramming of Adipocytes

### 2.1. Reciprocal Modulation of Metabolic Reprogramming and Orchestration of Organelle Homeostasis in Beige Adipocytes

Metabolic reprogramming in beige adipocytes can be enhanced by three core regulators: AMPK, PGC-1α, and Nrf2. AMPK and PGC-1α promote mitochondrial biogenesis and oxidative phosphorylation, whereas Nrf2 upregulates antioxidant enzymes to counteract reactive oxygen species (ROS) generated during sustained thermogenesis [14,15,16,17]. This equilibrium is essential for prolonged energy expenditure [18]. Additionally, shifting immune responses toward an anti-inflammatory Th2 phenotype (away from Th1) alleviates inflammation-driven suppression of beiging [19,20].

Sustained mitochondrial respiration increases electron flux through ETC complexes I and III, thereby increasing the production of ROS [21]. This oxidative pressure activates Nrf2, which induces cytoprotective genes (*nqo1*, *gclc*, *sod2*, and *prdx1*), thereby maintaining redox balance [22]. A key metabolic node linking the mitochondria to the ER is the diversion of α-ketoglutarate from the TCA cycle to glutamate synthesis [23]. Glutamate is the direct precursor of glutathione (GSH), the primary antioxidant that maintains endoplasmic reticulum (ER) redox homeostasis [24]. Thus, the mitochondria–ER axis preserves redox stability during the formation of beige adipocytes.

The ER manages the metabolic load through domain specialization; the smooth ER handles lipid metabolism, whereas the rough ER activates the unfolded protein response (UPR) to mitigate proteotoxic stress [25]. Building on this, the Golgi apparatus processes and sorts lipids and proteins for adipocyte remodeling [26]. Maintaining ER–Golgi homeostasis is critical for UCP1-independent thermogenesis (e.g., creatine cycling and calcium signaling) [27]. In contrast, canonical UCP1-dependent thermogenesis relies on the rapid expansion of the mitochondrial proteome and efficient nuclear–mitochondrial transport [28].

**Figure 1:**
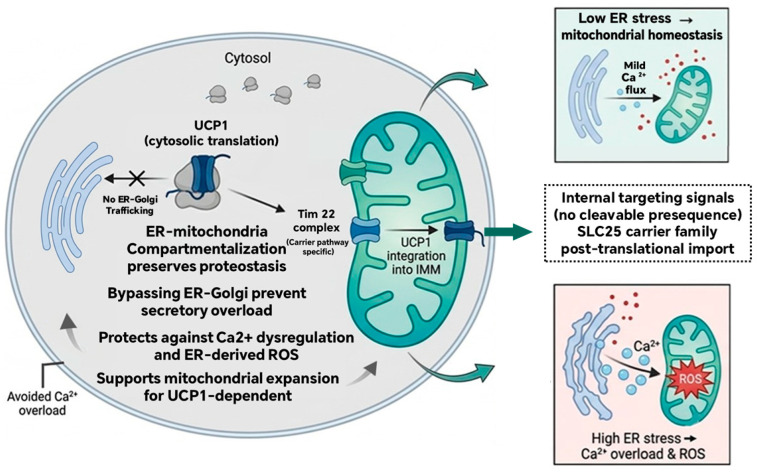
Posttranslational import of UCP1 and ER–mitochondrial proteostasis. UCP1 is synthesized on cytosolic ribosomes and imported directly into the inner mitochondrial membrane (IMM) via the TIM22 carrier translocase, bypassing the ER–Golgi pathway. This spatial segregation prevents ER luminal overload and ER-derived ROS, thereby preserving physiological Ca^2+^ flux. Low ER stress supports mitochondrial expansion and thermogenesis, whereas high ER stress leads to Ca^2+^ overload and ROS, which disrupt UCP1 function. The direct import of UCP1 is an evolutionary adaptation for high-flux thermogenesis.

UCP1 is imported directly into the inner mitochondrial membrane via TIM22 translocase, thereby bypassing the ER–Golgi pathway, a mechanistic safeguard (Figure 1) [29]. This process relies on internal targeting signals within its six-transmembrane bundle, shunting the canonical N-terminal cleavable pre-sequence [30,31]. Such spatial segregation prevents ER overcrowding, mitigates pathological Ca^2+^ efflux and ER-derived ROS, and shields mitochondrial matrix homeostasis [32,33,34].

### 2.2. Functional Rewiring of Major Canonical Metabolic Flux and Subcellular Architecture

#### 2.2.1. Reprogramming of Central Carbon Metabolism

Beiging induces profound reprogramming of canonical metabolic pathways, modulating lipid, carbohydrate, and protein metabolism to support the high energy demands of thermogenesis [35]. These adaptations sustain electron transport chain (ETC) activity even under post-inflammatory reactive oxygen species (ROS) conditions by maintaining tricarboxylic acid (TCA) cycle flux [36]. This is achieved through the coordinated regulation of citrate synthase (CS), isocitrate dehydrogenase (IDH), and oxaloacetate replenishment, which favors continued lipid catabolism while simultaneously promoting thermogenic energy expenditure [37,38,39].

#### 2.2.2. GLUT4-Mediated Glucose Uptake as a Metabolic Integrator

GLUT4-mediated glucose uptake provides a representative integrative axis that dynamically supplies carbon substrates from glycolysis to the tricarboxylic acid (TCA) cycle, a pivotal link to hypoxic and inflammatory cues for the metabolic reprogramming required [40]. One key effector of this adaptation is bidirectional lactate transport via MCT1, which exchanges lactate and related monocarboxylates to maintain redox balance [41]. Under hypoxia-inducible factor 1α (HIF-1α)-mediated hypoxia, glycolytic intermediates are routed toward lactate, but under permissive conditions, they undergo mitochondrial oxidation [42]. Enhanced pyruvate uptake fuels the TCA cycle through CS, and IDH, leading to continuous generation of oxaloacetate, ensuring the subsequent replenishment of NADH and FADH2. This coordination secures the electron supply to the ETC during thermogenesis and oxidative stress [43].

#### 2.2.3. Hypoxic Glycolytic Bypass and Lactate Production

Expansion of the adipose tissue results in local hypoxic conditions that trigger glycolytic bypass [44]. Glucose uptake via GLUT4 channels serves not only as an auxiliary fuel but also sustains anaerobic metabolism [45]. Glyceraldehyde-3-phosphate (G3P) is redirected toward lactate production via HIF-1α stabilization, which diverts pyruvate from mitochondrial oxidation to lactate formation through lactate dehydrogenase (LDH) [46]. This mechanism maintains glycolytic throughput when oxidative phosphorylation is constrained, ensuring a continuous supply of ATP and biosynthetic intermediates.

#### 2.2.4. Amino Acid Catabolism Integration in TCA and Proteostasis

Metabolic convergence is functionally coupled to protein turnover and mitochondrial biogenesis. Glutamate–ammonia ligase activity and branched-chain amino acid (BCAA) catabolism function as nitrogen-buffering systems in adipose depots [47,48]. By linking protein quality control to metabolic output, these pathways ensure that the energy-dissipative state of beige adipocytes remains structurally and metabolically sustainable. During thermogenesis, the expansion of the mitochondrial network increases the demand for carbon and nitrogen substrates. The BCAA–glutamate axis meets this demand by recycling nitrogen while supplying carbon precursors to the tricarboxylic acid (TCA) cycle [49]. Notably, beige adipocytes re-orchestrate the flow of carbon and nitrogen intermediates to act as bioenergetic sensors, preventing proteotoxicity during the rapid synthesis of the thermogenic proteome.

#### 2.2.5. Quality Control: Autophagy–Mitophagy Axis and Physiological Modulation

**Figure 2:**
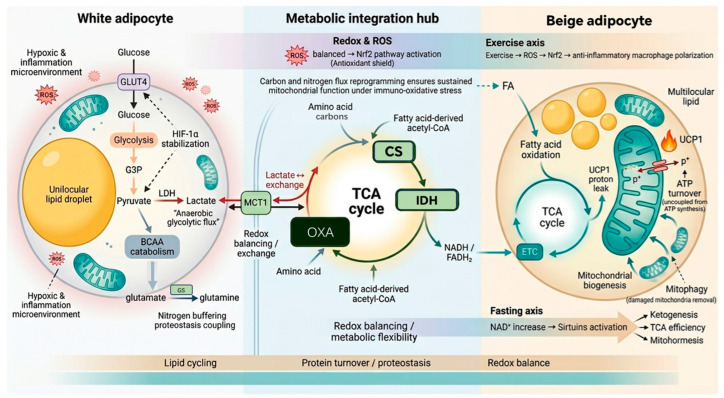
Metabolic and redox integration in white-to-beige adipocyte reprogramming. Under hypoxic/inflammatory stress, white adipocytes increase glucose uptake and lactate exchange via MCT1, while BCAA catabolism supplies nitrogen for proteostasis. Carbon and nitrogen fluxes converge on the TCA cycle. Citrate synthase (CS) condenses oxaloacetate (OXA) with acetyl-CoA, and isocitrate dehydrogenase (IDH) converts isocitrate to α-ketoglutarate, generating NADH/FADH_2_ for the electron transport chain. Balanced redox promotes mitochondrial biogenesis, ketogenesis, and TCA efficiency. Fasting (NAD^+^ → sirtuins) and exercise (ROS → mitophagy) enhance quality control. In beige adipocytes, UCP1-mediated proton leak uncouples ATP synthesis from the ETC, enabling thermogenesis while maintaining redox balance.

Mitochondrial quality is preserved through a specialized autophagic–mitophagic axis that selectively removes reactive oxygen species (ROS)-damaged organelles, which is a cytoprotective layer often overlooked in traditional beiging models [50]. This quality control system remains highly responsive to physiological modulators. Exercise-induced ROS activates the Nrf2-dependent antioxidant response, promoting the resolution of inflammation via macrophage polarization and enhancing fatty acid oxidation [51]. In parallel, fasting-induced increases in NAD^+^ levels activate sirtuin signaling, enhancing ketogenesis and refining the tricarboxylic acid (TCA) cycle efficiency for mitohormetic adaptation [52]. However, not all autophagic processes uniformly support thermogenesis; distinct pathways can exert opposing effects (Figure 2) [53]. Recent evidence indicates that while chaperone-mediated autophagy (CMA) sustains BAT thermogenic activity and declines with age, excessive macroautophagy is linked to whitening and age-related BAT dysfunction, suggesting that systemic autophagy induction may be counterproductive at least in mice models [54].

## 3. Inflammatory and Macrophage Mediated Suppression of Beige Adipocyte Thermogenesis

### 3.1. From Adipocyte Stress to Immune Reprogramming: Molecular Checkpoints Governing Beige Fat Decline

In obesity, pro-inflammatory cytokines are released from stressed adipocytes and M1 macrophages [55,56] act in a feed-forward loop: TNF-α directly suppresses energy expenditure (UCP-1 inhibition) and induces TGF-β production, with the latter effect mediated by ERK/AP-1 signaling [57,58], ultimately abrogating the beiging of white adipocytes [59]. TGF-β disrupts PPARγ–C/EBPα complex formation [60], and mice overexpressing TGF-β show an arrested beiging and lipodystrophic phenotype [61]. These cytokines do not act alone; they also prime the NLRP3 inflammasome. As a consequence, NLRP3 activation promotes M1 polarization and IL-1β secretion [62]. which downregulates UCP1 expression and impairs mitochondrial bioenergetics [63]. The activity of NLRP3 is tightly controlled by the autophagy adaptor p62/SQSTM1. Under normal conditions, p62 clears damaged mitochondria (mitophagy), reduces ROS, and restrains NLRP3. In obesity, p62 aggregates accumulate, enhancing NLRP3 transcription and assembly, and worsening inflammation. Notably, p62 forms condensates that sequester NLRP3 and ASC, acting as a brake under metabolic stress [64]. p62/p38 MAPK signaling further enhances UCP1 expression, and p62-deficient mice show impaired thermogenesis [65]. Thus, inflammatory cytokines (TNF-α and TGFβ) and the NLRP3 p62 axis are functionally linked, with cytokines triggering the inflammasome and p62 modulating its intensity.

### 3.2. Contextual Plasticity of M2 Macrophages in Adipose Thermogenesis

**Figure 3:**
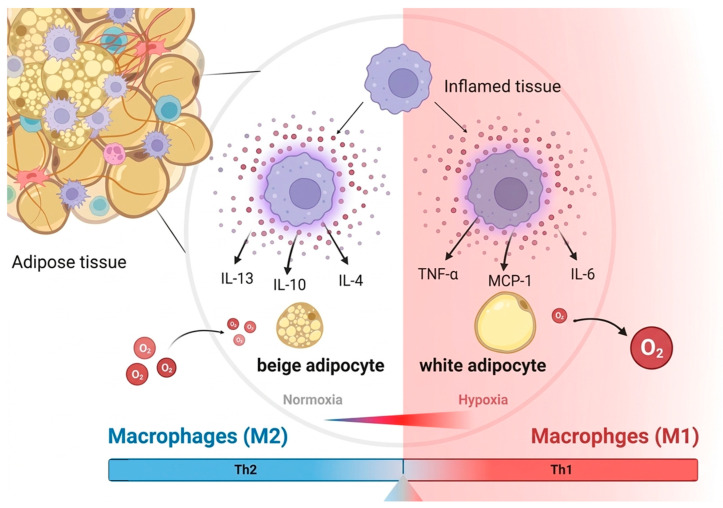
Comparison of the adipose tissue microenvironment under normoxia and hypoxia. Normoxia (**left**) promotes beige adipocytes and M2 macrophage polarization (Th2 response: IL-13, IL-10, IL-4). Hypoxia (**right**) induces an inflammatory state with white adipocyte development and M1 macrophage polarization (Th1 response: TNF-α, MCP-1, IL-6).

Beyond this pro-inflammatory cascade, the loss of M2-dependent support independently impairs the beiging process. M2 macrophages respond to adipocyte-derived adiponectin during cold exposure, and the loss of macrophages or adiponectin reduces cold-induced beiging [66]. Adrenomedullin 2 (ADM2) enhances UCP1 via cAMP/PKA–p38 MAPK and reactivates M2 macrophages, forming a positive feedback loop [67]. In brown adipose tissue, obesity shifts resident M2 macrophages toward M1 macrophages, thereby repressing UCP1 expression [68,69]. Macrophages do not produce catecholamines (they lack tyrosine hydroxylase), and sympathetic neurons are the primary source [70]. Nonetheless, the IL-13/IL-13Rα1 axis is critical, and its deletion impairs cold-induced beige adipocyte formation [71]. However, macrophages are not merely supportive bystanders; a distinct population of sympathetic neuron-associated macrophages (SAMs) in BAT actively limits thermogenesis. SAMs express the norepinephrine transporter Slc6a2 and monoamine oxidase-A, enabling them to take up and degrade neuronally released norepinephrine, thereby constraining adrenergic tone [72]. This direct neuro-immune crosstalk reveals a macrophage-dependent brake on thermogenesis that can be pathologically reinforced in obesity, adding an additional layer beyond the classical M1/M2 paradigm (Figure 3).

## 4. Hormetic Oxidative State, Autophagy, and Mitochondrial Dysfunction During Adipocyte Remodeling

### 4.1. Autophagy as a Metabolic Adaptation in Adipose Plasticity

Fasting-induced energy deprivation activates AMPK, which directly phosphorylates ULK1 to initiate autophagy [73]. This process facilitates the clearance of dysfunctional mitochondria and lipid droplets, thereby maintaining adipocyte function [74]. In obesity, the AMPK–ULK1–autophagy axis is often disrupted, contributing to metabolic dysfunction [75]. Autophagy ensures the selective turnover of damaged proteins and organelles (mitophagy and lipophagy), thereby preserving adipocyte homeostasis [76]. Dysregulated autophagy impairs lipid mobilization both in vivo and in vitro, contributing to resistance to weight loss [77]. ROS can initiate autophagy by modulating AMPK and mTOR; conversely, balanced autophagy mitigates oxidative stress by removing damaged organelles and oxidized intermediates [78].

Mitochondrial carrier homolog 2 (MTCH2) acts as an energy expenditure suppressor by negatively regulating autophagy via the Bcl-2 pathway and its crosstalk with PINK1/Parkin-mediated mitophagy, leading to the selective degradation of damaged mitochondria [79]. Pro-inflammatory cytokines modulate autophagic flux, whereas autophagy constrains inflammatory signaling, forming a bidirectional regulatory axis [78]. Metformin and vitamin D, for instance, disrupt autophagosome biogenesis and suppress inflammatory pathways, attenuating white adipogenesis while promoting thermogenesis in brown fat [80]. This tightly controlled turnover regulates the number and function of mitochondria available for thermogenic activation.

### 4.2. Therapeutic Targeting of Antioxidant Pathways During Adipose Beiging

**Figure 4:**
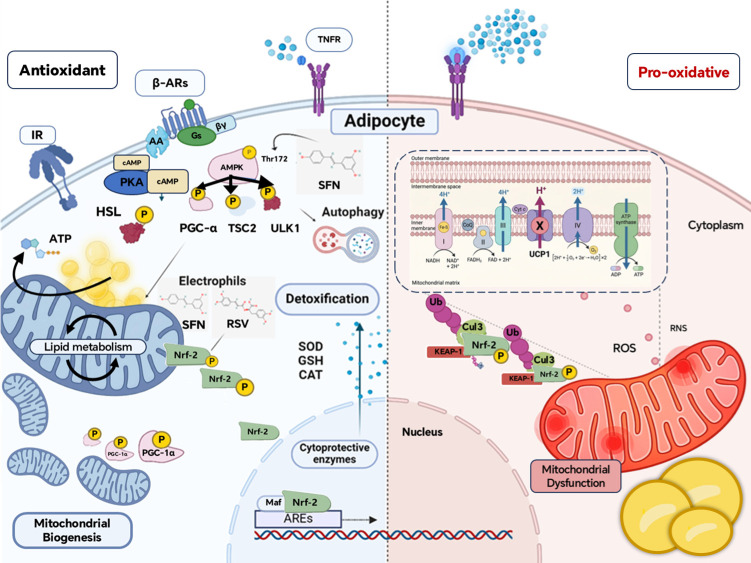
Orchestration of the redox–mitochondrial axis in adipocyte plasticity and thermogenesis. β-adrenergic signaling and electrophiles (SFN, RSV) activate AMPK (Thr172), promoting PGC-1α-dependent mitochondrial biogenesis and autophagy. Nrf2 dissociates from KEAP1, translocates to the nucleus, and induces ARE-driven antioxidant enzymes (SOD, GSH, and CAT), thereby preserving redox homeostasis. In contrast, excessive ROS/RNS enhances KEAP1/Cul3-mediated Nrf2 degradation, leading to mitochondrial dysfunction and thermogenesis impairment. UCP1-mediated H^+^ leakage uncouples oxidative phosphorylation from ATP synthesis to generate heat. Arrows indicate activation and/or phosphorylation of molecular flux.

Resolving the crosstalk between adipocyte dysfunction and thermogenesis relies on the mitochondrial-related redox axis [81]. This node integrates AMPK-mediated autophagy with antioxidant defense to ensure organelle integrity during a high respiratory flux. Naturally derived electrophiles (e.g., resveratrol and sulforaphane) boost the endogenous antioxidant capacity [82,83]. Figure 4 illustrates how β-adrenergic activation and dietary electrophilic signals converge on AMPK and Nrf2 to promote mitochondrial maintenance and neutralize mtROS [84]. Adipocytes maintain plasticity by balancing UCP1-mediated thermogenesis with ARE-driven cytoprotection [85].

Given the association between metabolism, oxidative stress, and immune profiles in humans and murine models, modulating antioxidant pathways during WAT transition is a compelling strategy for obesity treatment. The Keap1-Nrf2 axis master regulates ARE-driven gene expression that detoxifies ROS [86]. Upon release from Keap1/CUL3 repression, Nrf2 translocates to the nucleus, heterodimerizes with Maf proteins, and activates ARE-dependent transcription [87]. Nrf2 plays an integral role in lipid metabolism and oxidative defense in 3T3-L1 adipocytes and in mice [88,89]. Thus, the Keap1-Nrf2 pathway acts as a metabolic rheostat amenable to pharmacological exploitation to restore mitochondrial function and promote favorable adipose tissue remodeling.

ROS during thermogenic activation has dual implications: it acts as a second messenger to amplify PGC-1α and UCP-1 expression; however, unchecked ROS accumulation compromises mitochondrial integrity and adipocyte viability [90]. SOD2 protects beige adipocytes from oxidative stress; increased SOD2 elevates thermogenic output and resilience to a high-fat diet, suggesting SOD2 activation as an in vivo browning strategy [91]. Overexpression of mitochondrial catalase extends the lifespan of UCP1-expressing adipocytes during prolonged cold exposure [92]. Beige adipocyte thermogenesis crucially depends on GSH-driven GPX4 activity to prevent ferroptosis (iron-dependent lipid peroxidation cell death), positioning the cystine–GPX4 axis as a promising intervention for sustaining adipose redox balance and metabolic resilience under oxidative stress [93].

## 5. The Interplay of Molecular, Immune, and Oxidative Signaling in Adipocyte Beiging: Insights into Bioactive Compounds and Modulators of Adipocyte

Bioactive compounds can modulate organelle proteostasis and macrophage polarization; however, their clinical utility is frequently compromised by poor pharmacokinetics and lack of tissue-specific targeting [94]. The transition from the laboratory to the clinic is obstructed by a gap between molecular efficacy and physiological bioavailability; systemic distribution issues reduce adipose tissue accumulation and increase the risk of off-target effects [95,96].

Several compounds converge on the AMPK–SIRT axis to promote beige adipocyte formation. Berberine activates SIRT3 and AMPK/SIRT1, increasing PPARγ deacetylation and UCP1 levels while suppressing NLRP3/NF-κB inflammation in adipose macrophages [97,98,99]. Gingerol promotes lipolysis and mitochondrial biogenesis via AMPK activation in pre-adipocytes [100]. Quercetin acts through SIRT1 to deacetylate NF-κB p65, preventing its nuclear translocation and reducing TNF-α/IL-6 production in brown adipose tissue [101]. Collectively, these findings position the AMPK–SIRT network as a central node in the action of natural compounds.

The second group targets cAMP/PKA signaling. Isomeranzin directly binds Gnas (G(s)α), triggering a cAMP-AMPK cascade that drives WAT browning [102]. Naringenin enhances isoproterenol-stimulated UCP1 expression via PKA/p38 MAPK and PPARγ-dependent brown adipogenesis in 3T3-L1 cells [103]. Caffeine increases lipolysis and thermogenesis through cAMP/PKA activation in primary rat adipocytes, synergizing with epinephrine [104].

Antioxidant and anti-inflammatory pathways provide a third mechanistic cluster. Sulforaphane activates Nrf2/ARE, upregulating glucose uptake and fatty acid oxidation in skeletal muscle, thereby reducing adiposity and improving insulin sensitivity [105]. The CYP2E1 inhibitor Q11 lowers ROS production, restores AMPK/PGC-1α signaling, and improves mitochondrial fusion/fission balance [106]. Sulforaphane and Q11 primarily act by relieving oxidative stress, which is a prerequisite for maintaining mitochondrial integrity during beiging.

Despite these promising mechanisms, major translational barriers remain. Most rodent studies rely on male C57BL/6 mice housed at sub-thermoneutral temperatures (~22 °C), which induces chronic cold stress and artificially exaggerates beiging effects [107,108]. Reliance on 3T3-L1 pre-adipocyte lines and the absence of human depot-specific data further limit the adaptation to human heterogeneous depots [109]. Moreover, the pharmacokinetics of these compounds are unfavorable, as poor oral absorption and rapid first-pass metabolism often result in serum concentrations far below the therapeutic threshold in humans.

Future strategies must move beyond compound-by-compound promotion. None of these natural products match the efficacy of synthetic GLP-1 analogs (e.g., semaglutide), which act centrally on the brain to control appetite [110]. Targeted delivery systems, nanoparticle encapsulation, or adipose-homing peptides could deliver compounds directly to the mitochondria-ER interface at clinically relevant concentrations without systemic toxicity [111,112]. Until clinical trials address bioavailability, cold-stress artifacts, and human-mouse differences, these mechanisms remain high-potential frameworks rather than established therapies [113].

In summary, bioactive compounds activate beiging through three main axes—AMPK/SIRT, cAMP/PKA, and Nrf2/antioxidant; however, poor pharmacokinetics, chronic cold stress in animal models, and lack of human validation severely limit their translation. Targeted delivery and rigorous clinical testing are urgently needed (Table 1).

## 6. Conclusions

In light of the complexities of adipose tissue, a more nuanced approach that integrates redox signaling, immune remodeling, and metabolic rewiring is essential. Targeting through combinatorial phytochemical synergistic modulations may open new avenues for the treatment of metabolic diseases and insulin resistance. However, precision is key; interventions must be tailored to the adipose depot, inflammatory status, and insulin sensitivity to avoid paradoxical treatment effects. For instance, coumarin, quercetin, and berberine, which act as both metabolic realigners and redox buffers, hold promise not only for weight management but also for restoring adipose metabolic flexibility and insulin responsiveness. In conclusion, the therapeutic restoration of adipose metabolic flexibility requires immune and metabolic remodeling. By unifying the AMPK-PGC-1α-UCP1 signaling thread with Nrf2-driven antioxidant programs, it is possible to harness the thermogenic potential of beige adipocytes while mitigating the deleterious effects of obesity-induced oxidative stress and M1 macrophage infiltration. However, significant translational barriers remain, particularly regarding the species-specific divergence between murine UCP1-dependent mechanisms and human complex metabolic regulation, as well as the low bioavailability of bioactive compounds, such as quercetin and berberine. Future research must prioritize humanized models and spatial transcriptomics to define the temporal ROS thresholds necessary for signaling without inducing mitochondrial dysfunction. Ultimately, targeting this triad through precision pharmacology or combinatorial phytochemical modulation holds promise for overcoming cellular memory and restoring systemic insulin responsiveness in metabolic disease.

## Figures and Tables

**Table 1:** Key molecular targets and functional outcomes of bioactive compounds in adipose beiging.

Compound	Model System	Key Molecular Targets & Direction of Regulation (↑/↓)	Functional Outcome in Beiging/Thermogenesis	Ref.
Berberine	HFD-fed C57BL/6 mice, *O. niloticus*	SIRT3/AMPK ↑, SIRT1 ↑, PPARγ deacetylation ↑, NF-κB p65 deacetylation ↓, NLRP3 inflammasome ↓	Increases UCP1 in WAT; suppresses M1-like inflammation; enhances M2 polarization	[99,114,115]
Isomeranzin	HFD-fed mice (i.p. 50 mg/kg)	Gnas (Gsα) ↑ → cAMP ↑ → AMPK ↑	Drives WAT browning; improves systemic insulin sensitivity	[102]
Naringenin	3T3-L1 adipocytes	PKA/p38 MAPK ↑, PPARγ ↑	Enhances isoproterenol-stimulated UCP1 expression; promotes brown adipogenesis	[103]
Sulphoraphane	HFD-fed male C57BL/6 mice	Nrf2/ARE pathway ↑, Glucose uptake/FA oxidation ↑	Reduces adiposity; improves insulin sensitivity in skeletal muscle	[116]
Quercetin	HFD-fed male mice (100 mg/kg)	SIRT1 ↑ → NF-κB p65 deacetylation ↓ → TNFα/IL-6 ↓	Alleviates BAT inflammation; preserves mitochondrial integrity	[101]
Q11 (CYP2E1 Inhibitor)	HFD-fed male mice (10–40 mg/kg)	CYP2E1 ↓, ROS ↓, AMPK/PGC-1α ↑	Restores mitochondrial fusion/fission balance; alleviates adipose inflammation	[106]
Gingerol	Pre-adipocyte from inguinal white adipose depot (6 µL/mL)	AMPK ↑	Promote lipolysis, mitochondrial biogenesis	[100]
Caffeine	Primary rat adipocytes	cAMP/PKA ↑, synergizes with epinephrine	Increased lipolysis, and thermogenesis, decreased adipose inflammation	[104]

Arrows indicate the direction of change: ↑ = increase, upregulation, or activation; → = leads to, activates; ↓ = decrease, downregulation, or inhibition.

## Data Availability

No new data were created or analyzed in this study. Data sharing is not applicable to this article.

## Abbreviations

AMPK	AMP-activated protein kinase
AP-1	Activator protein 1
ASC	Apoptosis-associated speck-like protein containing a CARD
ARE	Antioxidant response element
ATP	Adenosine triphosphate
BAT	Brown adipose tissue
Bcl2	B-cell lymphoma 2
CAT	Catalase
Cul3	Cullin 3
CREB	cAMP response element-binding protein
ER	Endoplasmic reticulum
ERK	Extracellular signal-regulated kinase
ETC	Electron transport chain
FAO	Fatty acid oxidation
GPX4	Glutathione peroxidase 4
GSH	Glutathione
H+	Hydrogen ion
HSL	Hormone-sensitive lipase
IL	Interleukin (e.g., IL-1β, IL-4, IL-6, IL-13)
iNOS	Inducible nitric oxide synthase
Keap1	Kelch-like ECH-associated protein 1
LDH	Lactate dehydrogenase
MAPK	Mitogen-activated protein kinase
MCP-1	Monocyte chemoattractant protein-1
M1/M2	Macrophage type 1/type 2
Maf	Musculoaponeurotic fibrosarcoma oncogene homolog
MTCH2	Mitochondrial carrier homolog 2
MTS	Mitochondrial targeting sequence
NADPH	Nicotinamide adenine dinucleotide phosphate (reduced form)
NF-κB	Nuclear factor kappa-light-chain-enhancer of activated B cells
NLRP3	NOD-like receptor family, pyrin domain containing 3
Nrf2	Nuclear factor erythroid 2–related factor 2
OXPHOS	Oxidative phosphorylation
PGC-1α	Peroxisome proliferator-activated receptor gamma coactivator 1-alpha
PINK	PTEN-induced kinase 1
PKA	Protein kinase A
PPARγ	Peroxisome proliferator-activated receptor gamma
PPP	Pentose phosphate pathway
PRDM16	PR domain containing 16
Prdx	Peroxiredoxin
ROS	Reactive oxygen species
RNS	Reactive nitrogen species
RSV	Resveratrol
SFN	Sulforaphane
SIRT	Sirtuin (e.g., SIRT1, SIRT3)
Slc6a2	Human solute carrier family 6 member 2 gene
SOD2	Superoxide dismutase 2
SMAD3	Mothers against decapentaplegic homolog 3
SQSTM1/p62	Sequestosome 1
TCA cycle	Tricarboxylic acid cycle
TOM	Translocase of the outer membrane
TIM	Translocase of the inner membrane
TGFβ	Transforming growth factor beta
Th1/Th2	T-helper cell type 1/type 2
TNF-α	Tumor necrosis factor-alpha
Trx2	Thioredoxin 2
UCP1	Uncoupling protein 1
ULK1	Unc-51 like autophagy activating kinase 1
UPR	Unfolded protein response
WAT	White adipose tissue
